# Event and Comment

**Published:** 1912-04-15

**Authors:** 


### DENTAL REGISTER.

Vol. LXVI. · April 15, 1912. No. 4

### EVENT AND COMMENT.

COMMERCIAL COMPENSATIONS AND PROFESSIONAL REWARDS. In these days of discussion as to ways and means for making the practice of dentistry financially compensating, the methods employed by people engaged in the barter or trade occupations have been so extensivly quoted and recommended as a remedy for our unbusinesslike practices, that there is grave danger that we may loose sight of the fundamental ideas which differentiate a professional from a commercial transaction.

In the first place it is an easy matter to place values on commercial transactions involving merely things. Things as a rule are the creations of man and can be duplicated at his pleasure and in practically unlimited quantities. In most cases they can be utterly destroyed without serious loss or harm, as they can always be replaced. Their monetary values are wholly fictitious and for the most part inherent. In other words there is nothing vital or so consequential in their existence as to make their entire loss of irreparable consequence. It naturally follows then that we are not seriously disturbed because the Beef Trust raises the prices of our meats, or the railroads boost the coal prices, so long as they do not set these values so high that human suffering and injury prevails to a considerable extent. We do not therefor consider it a great crime that men combine themselves together in the interest of trade, even though we know they do so that they may secure for themselves a larger share of this world's material things than their neighbors.

But suppose the doctors and dentists conclude to make a combine for monetary advantage, wouldn't there be a great disturbance in our social and civic relations? Could we apply the present day business methods to professional activities without causing a revolution? Why not? In the first place there is a vital interest entering as the important factor in our problem, which the people would never consent to place over against any monetary values, because of their notions of the sacredness of life. Life, health and freedom are the great essentials of happiness, and these can never be bought nor sold, neither will the people consent willingly and knowingly to a traffic in them. Therefore it is impracticable for any one to place on them any sort of a commercial rating.

But some one will say that we do not propose to buy or sell our patients, all we propose to do is to "*sell our services.*" This phrase is the motto of the new campaign, but wherein this can be shown to be free from trading in health and happiness, and possibly life itself, is not quite so clear.

In old times the medical man was not paid by the job or even at the rate of so much for each visit or ministration. He was given every opportunity to rescue life and according to the measure of his success he was honored and rewarded; not always or even often in monetary values, but his skill and ministry were deified; as witness the old Greek healer Esculapius, who was made one of their greatest gods, and to him they built shrines and temples. In our day, whom do we honor more than the trusted family doctor? There have been, and there are mercenary doctors; but no honest man can practice medicine for a long period without being impressed with the spirit of whole hearted and altruistic service. An acquaintance of ours practiced medicine in a large city, and was called one night to see a dying babe in a deserted building with no one except a devoted mother to care for it. It was the terrible diptheria and the child could not long endure the struggle unless the phlegm could be removed from its air passages. No medicines could act soon enough, so the physician, wholly conscious of his own risk, placed his mouth over the little one's and cleared the child's throat and succeeded in staying the inevitable consequences until his medicines had time to take effect. Could there be anything more heroic, and could any one compensate with mere money such a ministration? When that physician went to make his charge for that visit does any one suppose he counted the cost he might have incurred by that one act? He never was compensated but he has his reward. Now the issues of the dental occupation are not so vital, but who knows how many lives are year by year hopelessly deprived of their rightful destinies because of a lack of proper dental ministrations at the right time; or who knows how many operations hurriedly or inadequately done because of the monetary influence have been the source of serious nervous and other physical disorders which have robbed people of their very chance for life and happiness.

We do not want here to say that good business methods are not necessary in the practice of our profession, because they are essential so long as society is organized on its present basis. What we are contending for is that we shall rise to that high plane of professional service that shall compel us to consider first of all our privileges as dental healers,—with a calling inferior to none,—which can only be realized when we have performed our whole duty in a purely altruistic and professional spirit. The rewards can then be adjusted on a basis of equity and justice if need be, or on the fairer basis of professional reward preferably.

